# Supplementary material for: Designer Small-Molecule Control System Based on Minocycline-Induced Disruption of Protein–Protein Interaction
Source: ACS Chem Biol. 2024 Jan 20;19(2):308–24. doi: 10.1021/acschembio.3c00521 (PMC10877577; doi:10.1021/acschembio.3c00521)
Supplement: Supplementary file 1 — cb3c00521_si_001.pdf [file cb3c00521_si_001.pdf]

Supporting information

Designer small molecule control system based on Minocycline induced disruption of protein-protein interaction.

AUTHORS

Ram Jha[1,2,3], Alexander Kinna[1,3], Alastair Hotblack[2], Reyisa Bughda[1], Anna Bulek[1], Isaac Gannon[1], Tudor Ilca[1], Christopher Allen[1], Katarina Lamb[1], Abigail Dolor[1], Ian Scott[1], Farhaan Parekh[1], James Sillibourne[1], Shaun Cordoba[1], Shimobi Onuoha[1], Simon Thomas[1], Mathieu Ferrari[1], Martin Pule*[1,2]

Affiliations

[1]Autolus Therapeutics, London, W12 7FP, UK.

[2]University College London, Research Department of Haematology, UCL Cancer Institute, London, WC1E 6DD, UK

[3]These authors contributed equally

*Correspondence to: m.pule@autolus.com

Figure S1. Chemical conjugation of minocycline

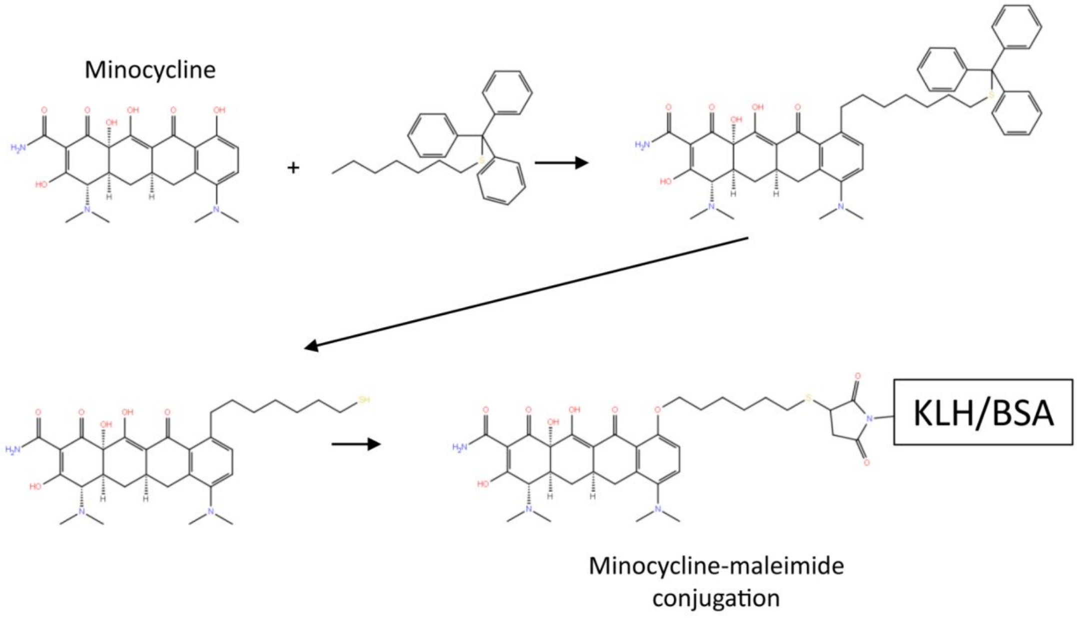

Functionalization of minocycline by addition of a free sulfhydryl group on a spacer arm to enable maleimide conjugation to either (KLH) or (BSA) for immunization and subsequent panning strategies, respectively.

Figure S2. Alpaca immunization and phage display output

A

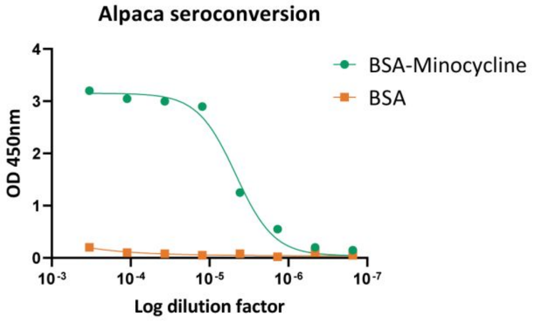

B

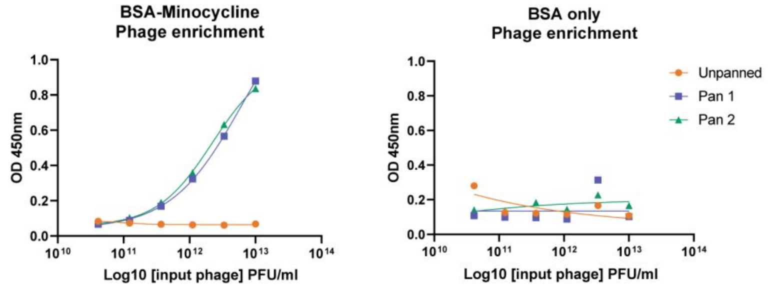

C

Clone	CDR 1	CDR 2	CDR 3
**aMinoC1**	GRTFSSYN	ISWSGART	AAGRGWGTEAILDY
**aMinoC4**	GRSLSSYV	ISWSGART	AAGRGWGTEAILDY
**aMinoC9**	GRTFSSYN	ISWSGART	VAGRGWGTEAILDY
**aMinoC14**	GRTFSNYN	INWSGGRT	AAGRGWGTEAILDY
**aMinoC21**	GRTFSSYN	ISWSGART	AAGRGWGTEAILDY
**aMinoC22**	GRTFSRYN	ISWSGART	AAGRGWGTEAILDY
**aMinoC25**	GRTFSSYN	ISWSGART	AAGRGWGTEAILDY
**aMinoC26**	GRTFSSYN	ISRSGGIT	AAGRGWGVEAILDY
**aMinoC31**	GNIGLVSV	ITGGGST	RLVNNGRPF
**aMinoC32**	GRLSLSSYV	ISWSGART	AAGRGWGTEAILDY
**aMinoC36**	GRTFSNYN	INWSGGRT	AAGRGWGTEAILDY
**aMinoC37**	GRTFSSYN	ISWSGART	AAGRGWGTEAILDY
**aMinoC38**	GRTFSSYN	ISWSGART	AAGRGWGTEAILDY
**aMinoC40**	GRTFSSYN	ISWSGART	AAGRGWGTEAILDY

D

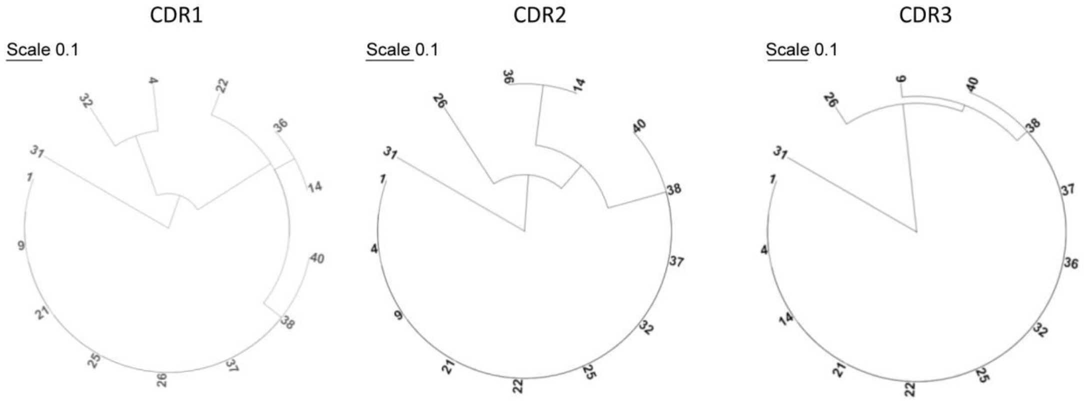

(A) Immunised Alpaca serum showing specific antibody response to minocycline in ELISA against immobilised BSA-minocycline (green) or BSA only (orange), detected with anti-alpaca IgG-HRP conjugated secondary antibody. **(B)** Minocycline specific phage enrichment ELISA for Pan 0 (unpanned, orange), Pan 1 (blue) and Pan 2 (green) against BSA-minocycline (left) and BSA only (right). Bound phage clones detected with anti-M13-HRP conjugated secondary antibody. **(C)** Complementarity-determining region (CDR) amino acid sequence analysis of phage derived anti-minocycline sdAb antibodies. **(D)** Phylogenetic divergence of CDR1, CDR2 and CDR3 in selected anti-minocycline clones.

Figure S3. SPR kinetic affinity of anti-minocycline sdAb

A

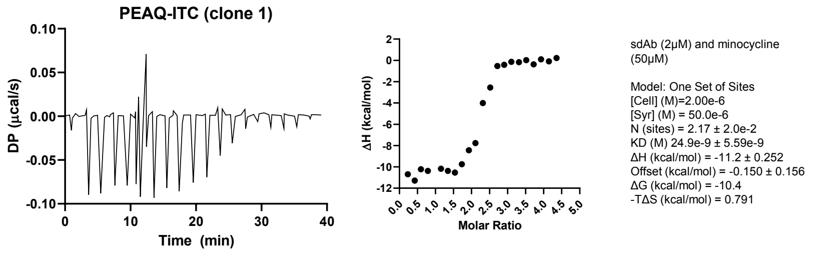

B

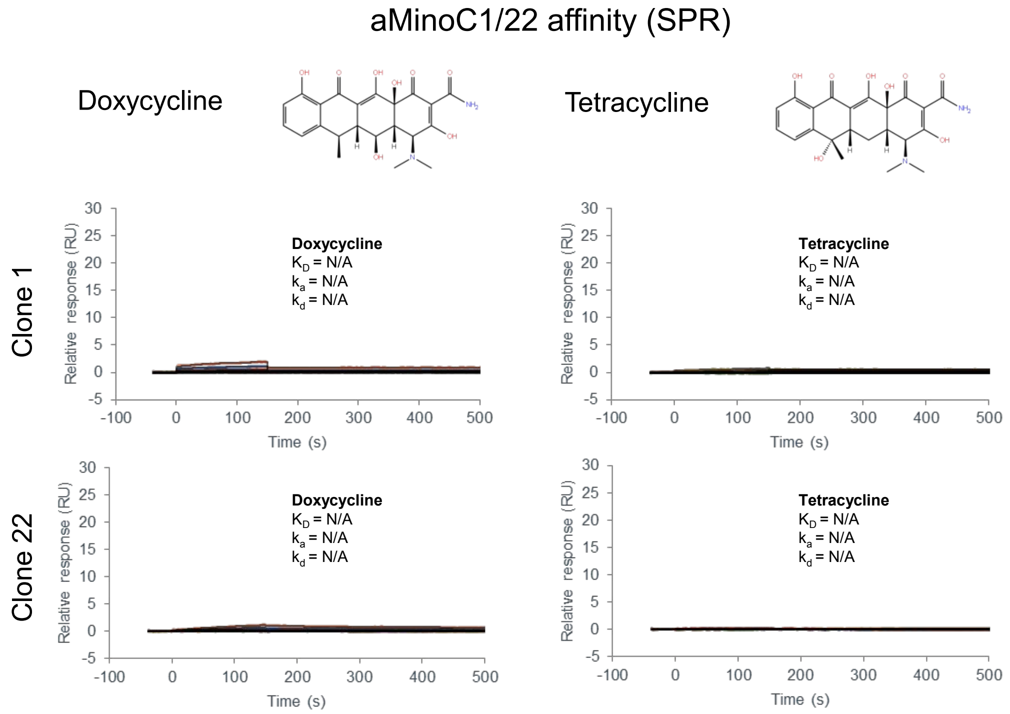

C

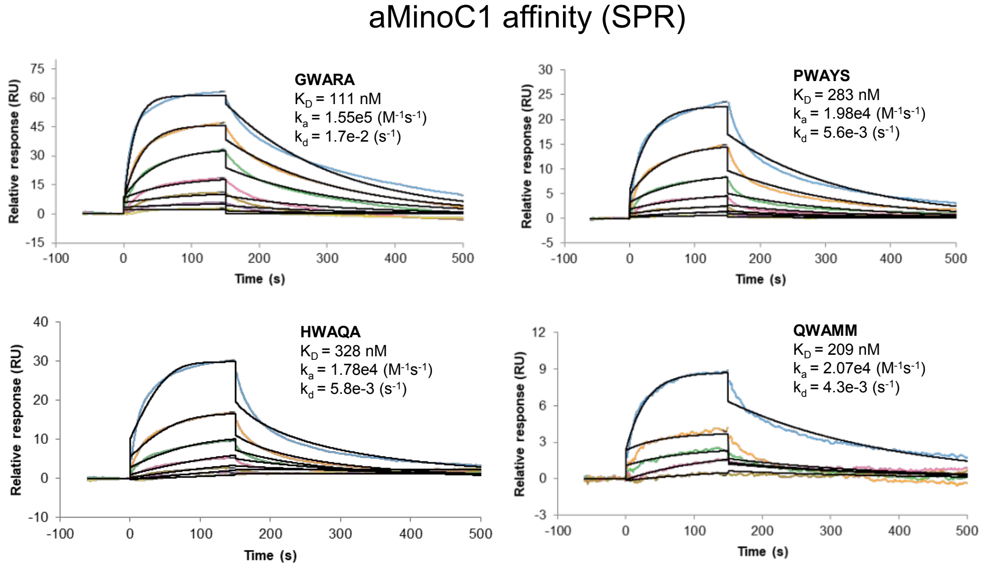

(A) Isothermal titration calorimetry (ITC) thermogram of aMinoC1 binding to minocycline. Raw thermogram plot over time showing ligand injection and binding saturation (top); and binding isotherm plot using one set of sites (1:1) binding model (Origin's software) showing minocycline/aMinoC1 molar ratio and enthalpy change of the reaction (bottom) which was used to deduce a KD value of 24.9 nM (± 5.59 nM). **(B)** Surface plasmon resonance (SPR) of aMinoC1 and aMinoC22 sdAb-Fc clones against doxycycline (left) and tetracycline (right) showing lack of binding to structurally related molecules to minocycline. **(C)** SPR kinetic affinity of GWARA, PWAYS, HWAQA and QWAMM peptide-Fc against aMinoC1. Kinetics fit with a 1:1 Langmuir binding model. Affinities were measured at 111 nM (GWARA), 283 nM (PWAYS), 328 nM (HWAQA) and 209 nM (QWAMM).

Figure S4. Dynamic binding of aMinoC1-CX$_7$C peptide-minocycline

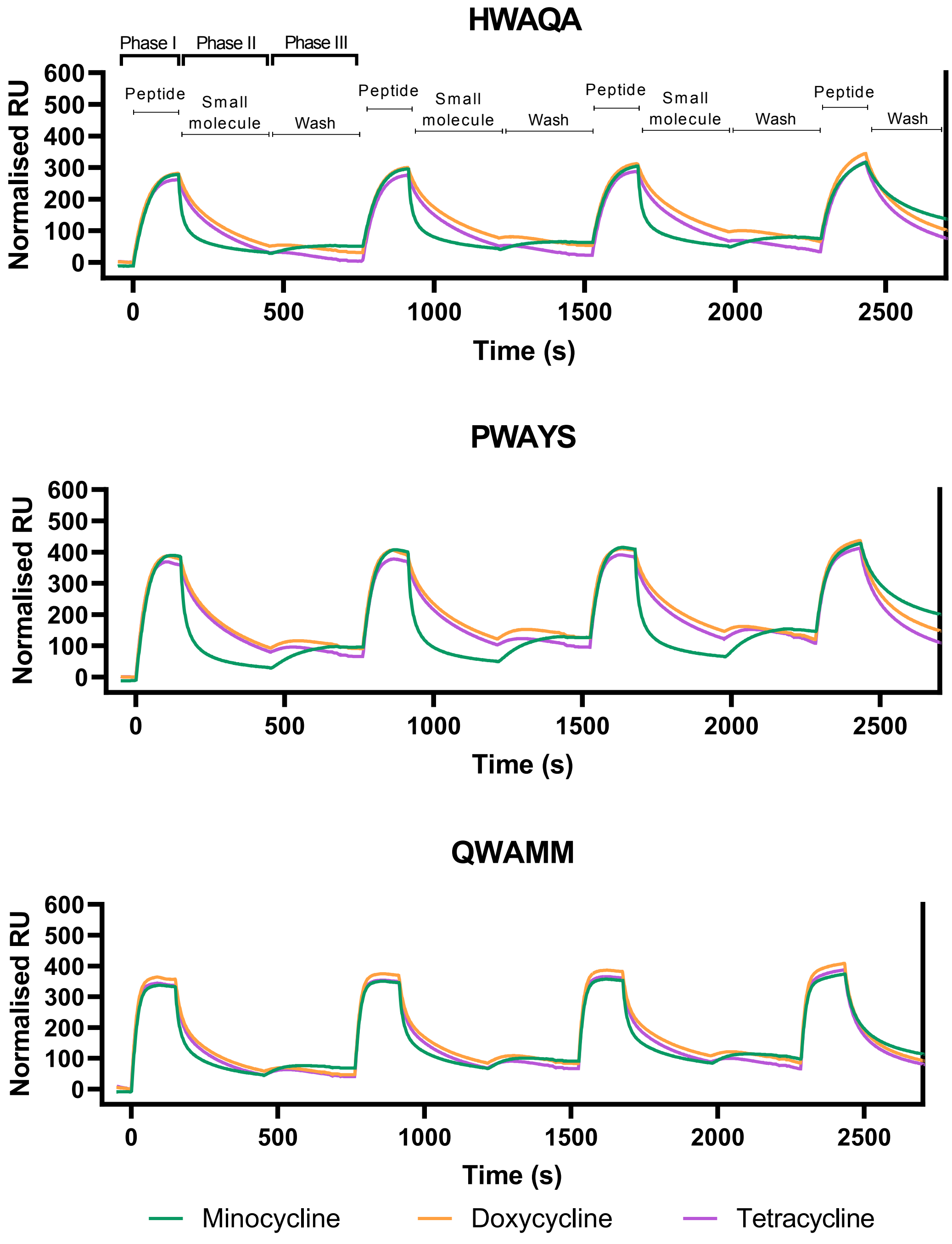

Dynamic minocycline (green), doxycycline (orange) or tetracycline (purple) small molecules and peptide-Fc (HWAQA, PWAYS and QWAMM) binding to immobilised aMinoC1 sdAb on Biacore T200. Sequential injections of GWARA-Fc (Phase I), small molecule (Phase II) and dissociation (buffer) step (Phase III) showing minocycline-driven acceleration of peptide-Fc dissociation. Serial challenges with peptide and small molecule show reversibility of the system. No enhanced dissociation visible with doxycycline or tetracycline injections.

Figure S5. Minocycline ligand preparation

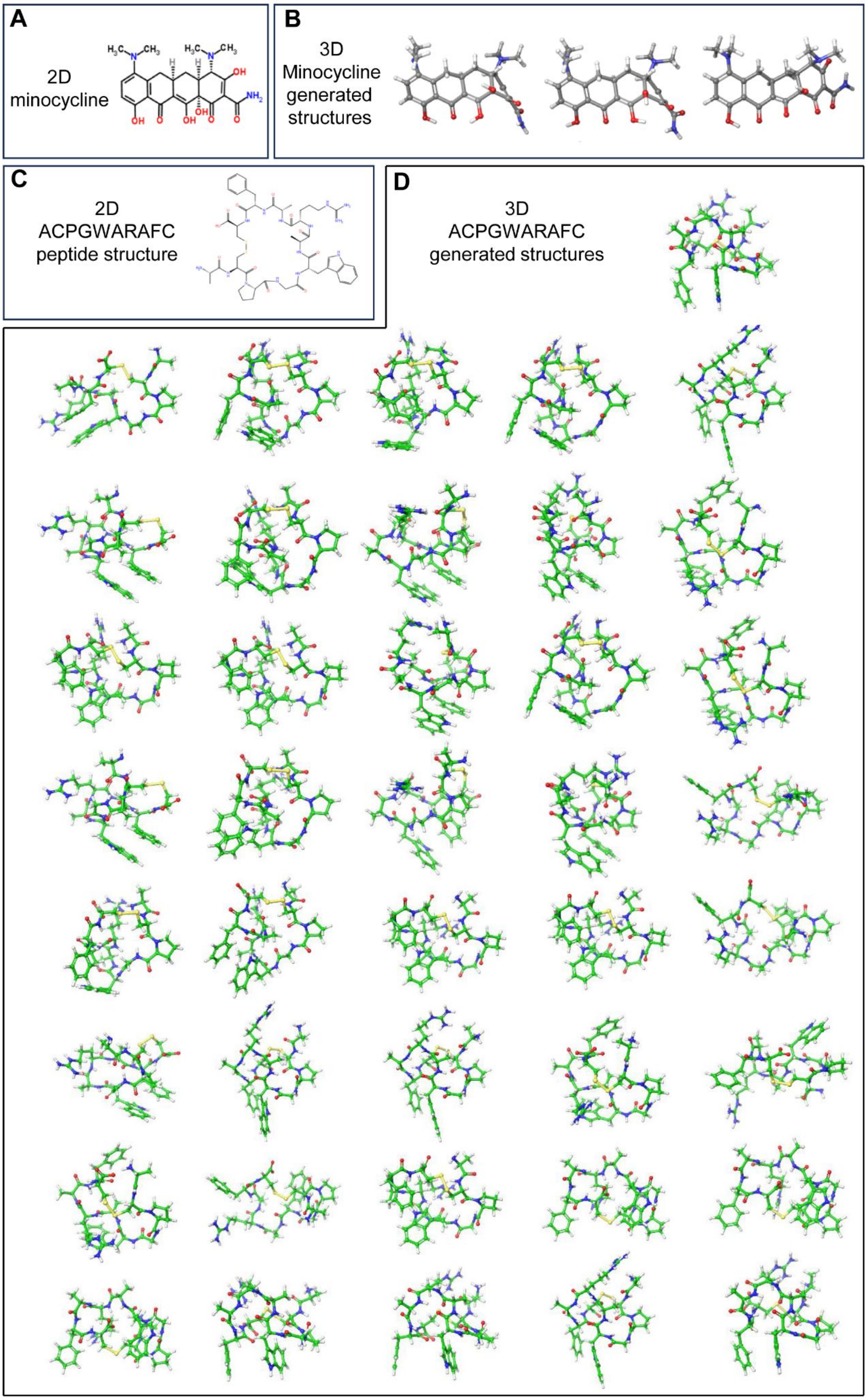

(A) 2D minocycline structure. **(B)** Energy minimised 3D structures for minocycline generated by Schrodinger LigPrep suite. **(C)** 2D cyclic GWARA (ACPGWARAFC) peptide structure. **(D)** Energy minimised 3D structures for GWARA peptide generated by Schrodinger LigPrep suite.

Figure S6. *In silico* immunogenicity analysis

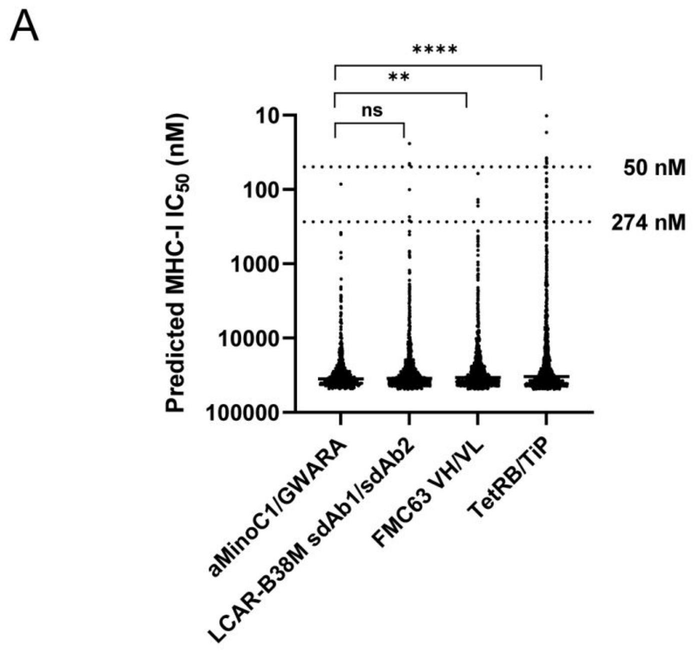

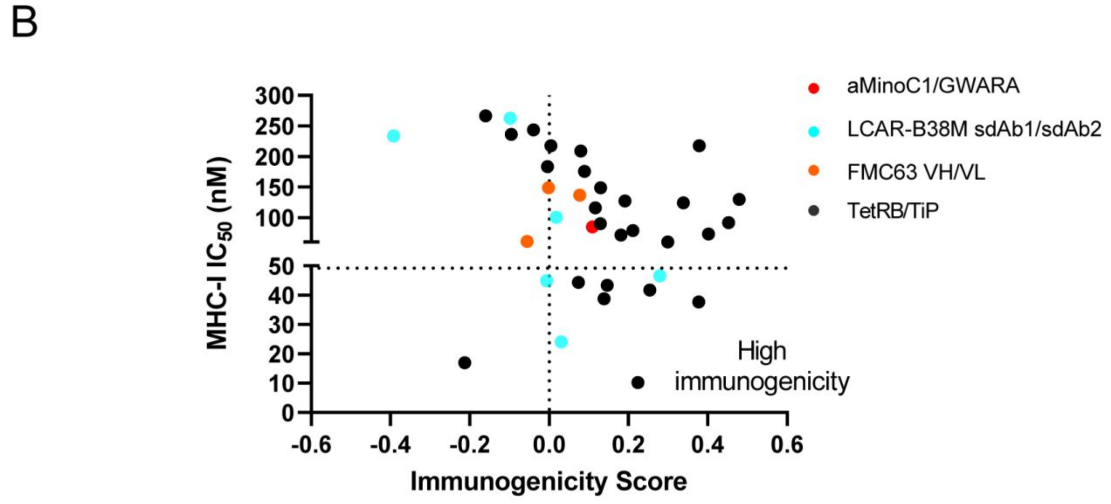

(A) Predicted MHC binding affinity for 9-14mer peptides derived from aMinoC1 and GWARA peptide, TetRB/TiP, FMC63 VH/VL and LCAR-B38M sdAbs. Affinity calculated using IEDB prediction tool. Dotted lines indicate cut-off for IC50 affinities of 274 nM (threshold for MHC-I engagement) and 50 nM (threshold for immune activation). Significantly lower IC50 affinities for aMinoC1/GWARA compared to bacterial TetRB/TiP and murine FMC53 VH/VL. One-way ANOVA with Dunnett's post-test, ** P <0.01, **** P <0.0001. **(B)** MHC-I IC_{50} and immunogenicity score for 9-14mer peptides from aMinoC1/GWARA (red), LCAR-B38M (teal), FMC63 VH/VL (orange) and TetRB/TiP (black) with < 274 nM MHC-I IC_{50} (from **A**). Positive score denotes potential immunogenicity. Peptides with < 50 nM MHC-I IC_{50} and positive immunogenicity score are considered highly immunogenic. No highly immunogenic peptides predicted for aMinoC1/GWARA.

Figure S7. Characterization of EGFR^{+} cell lines and MinoCAR cytotoxicity

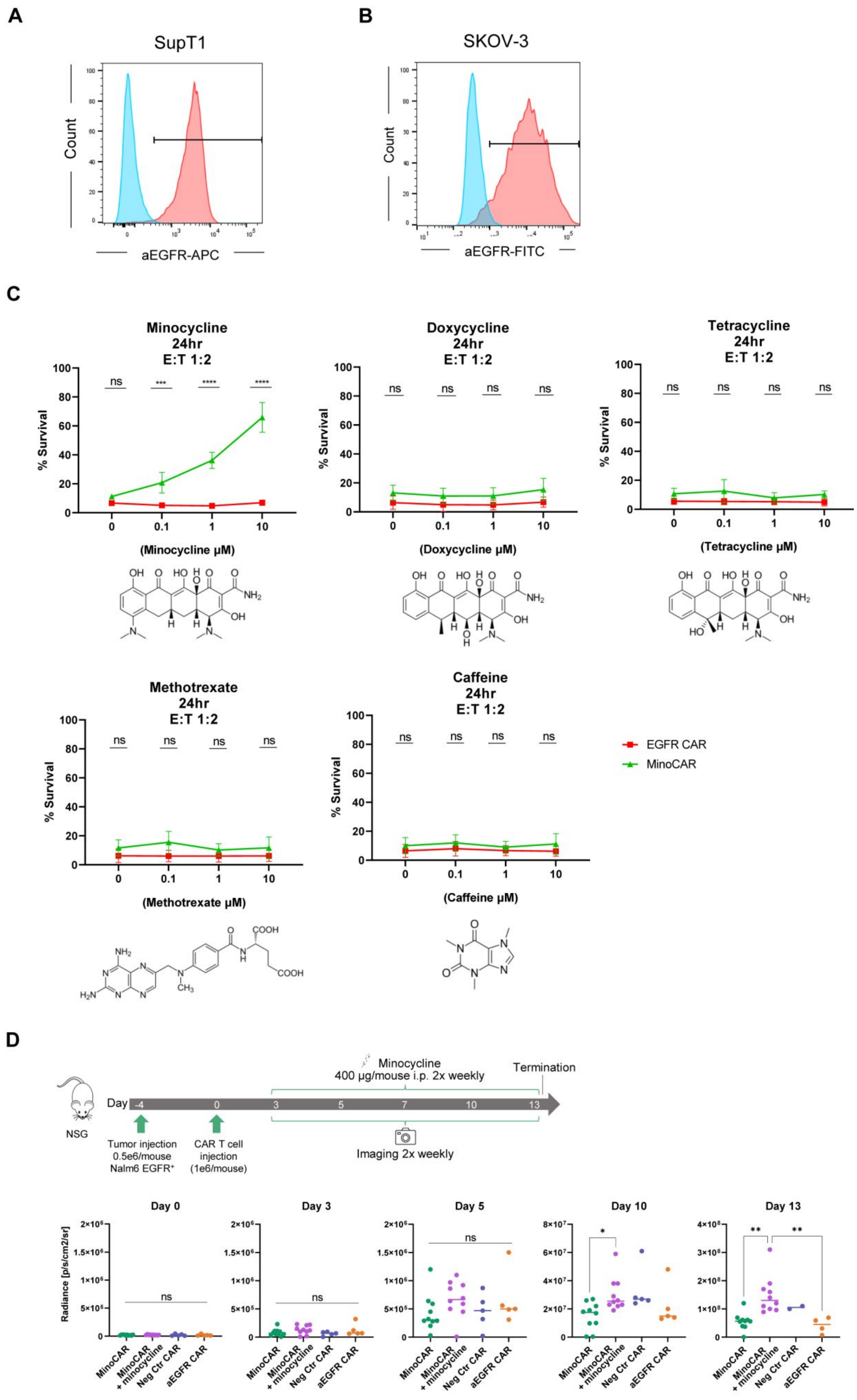

(A) SupT1 (blue) and SupT1 $EGFR^+$ cells (red) cells stained with anti-EGFR-APC antibody to confirm expression of EGFR marker. Histogram plot of cell count vs EGFR expression (MFI). **(B)** SKOV-3 mKate+ cells stained with anti-EGFR-APC to detect natively expressed EGFR (red). Unstained cells presented in blue. Histogram plot of cell count vs EGFR expression (MFI). **(C)** Flow cytometry-based cytotoxicity assay of PBMCs transduced with MinoCAR (green) and a conventional anti-EGFR CAR positive control (EGFR-CAR, red). Sup-T1 cells expressing EGFR (SupT1-EGFR+) were used as target cells. Effector cells were co-cultured with target cells at a ratio of 1:2 with varying concentrations of the indicated drug (0.1 μM, 1 μM and 10 μM) for 24 hours. The percent of live target cells was normalized to negative control. Gating strategy was as follows: Singlets (FSH/FSA)>Live cells (SYTOX blue)>Anti-CD3-PeCy7. Data represented as mean ± SD, n=4, two-way ANOVA with Sidak's post-test, *** P <0.001, **** P <0.0001. **(D)** (Top) Schematic of NSG Nalm6 EGFR+ tumor mouse model treated with MinoCAR (n=10), aEGFR CAR (n=5) or a negative control CAR carrying the SG_3S peptide (n=5). An additional cohort of MinoCAR included 400 μg/mouse twice weekly i.p. injections of minocycline (n=10). (Bottom) BLI readouts for CAR treated cohorts at days post car injection (DPI) 0, 3, 5, 10 and 13. DPI 7 is reported in Figure 4F. A significant difference in tumor burden control was detected for MinoCAR and MinoCAR + minocycline cohorts at DPI 7-13. One-way ANOVA with Tukey's post-test, * P < 0.05, ** P <0.01, ns = not significant. 3 mice from the negative control CAR and 1 from the eGFR CAR cohorts were sacrificed on day 11 for reaching the stated humane endpoint.

Figure S8. Dose response of GWARA-EGFR sdAb adaptor to UniMinoCAR

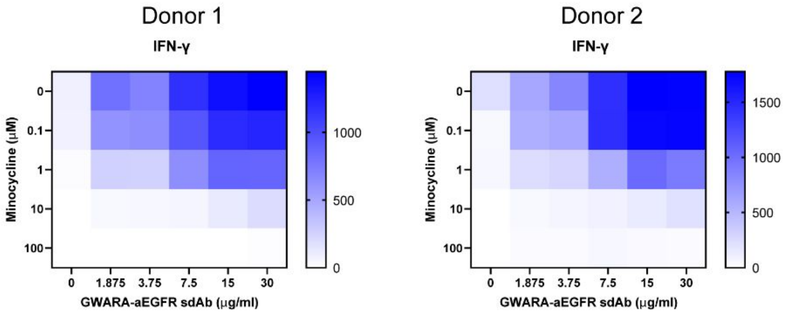

Dose response titration for GWARA-EGFR sdAb adaptor (from 0 to 30 μg/ml) in the presence of 0 to 100 μM minocycline. IFN-γ secretion measured from EGFR plate-based stimulation of transduced PBMCs with MinoUniCAR construct in each matrix condition. Two independent donors analysed.

Figure S9. Induced cell-cell interaction with aMinoC1/GWARA module

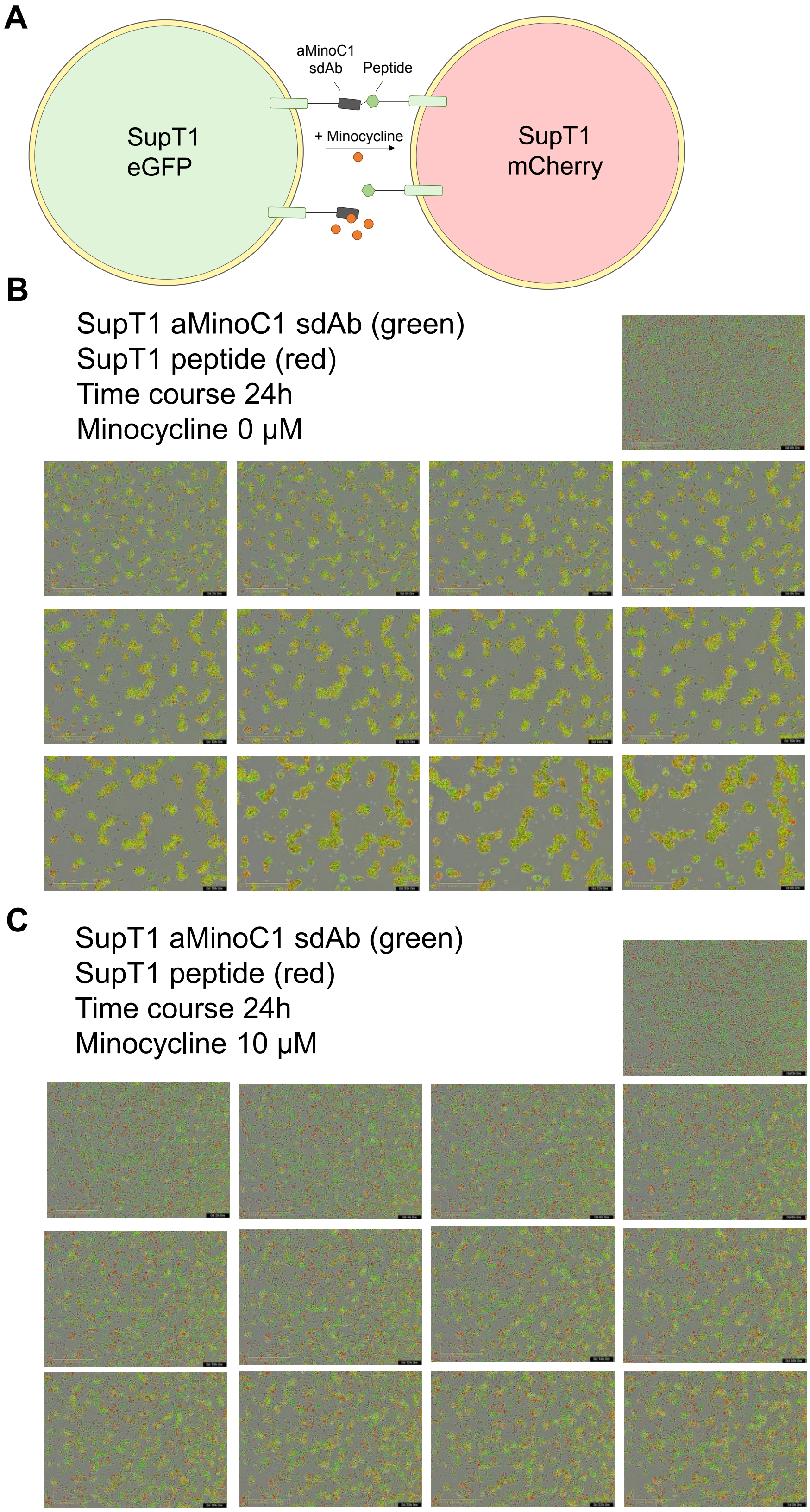

(A) schematic representation of SupT1 cells transduced with GWARA-CD8stk-CD28TM and mCherry (red) and SupT1 cells transduced with aMinoC1-CD8stk-CD28TM and eGFP (green). **(B)** Time course co-cultures of SupT1 GWARA-CD8stk-CD28TM mCherry (red) and SupT1 with aMinoC1-CD8stk-

CD28TM eGFP (green) for 24h in the absence of minocycline. Formation of aggregates is visible during co-culture. **(C)** Time course co-cultures of SupT1 GWARA-CD8stk-CD28TM mCherry (red) and SupT1 with aMinoC1-CD8stk-CD28TM eGFP (green) for 24h in the presence of 10 μM minocycline. Images acquired with IncuCyte at 2h intervals, representative of 3 independent experiments.
